# Rotary Jet Spinning (RJS): A Key Process to Produce Biopolymeric Wound Dressings

**DOI:** 10.3390/pharmaceutics14112500

**Published:** 2022-11-18

**Authors:** Juliana O. Bahú, Lucas R. Melo de Andrade, Sara Crivellin, Nadia G. Khouri, Sara O. Sousa, Luiza M. I. Fernandes, Samuel D. A. Souza, Luz S. Cárdenas Concha, Maria I. R. B. Schiavon, Cibelem I. Benites, Patrícia Severino, Eliana B. Souto, Viktor O. Cárdenas Concha

**Affiliations:** 1 INCT—BIOFABRIS, School of Chemical Engineering, University of Campinas, Albert Einstein Ave., Cidade Universitária Zeferino Vaz, nº. 500, Campinas 13083-852, São Paulo, Brazil; 2 Laboratory of Pharmaceutical Technology, Faculty of Pharmaceutical Sciences, Food and Nutrition, Federal University of Mato Grosso do Sul, Campo Grande 79070-900, Mato Grosso do Sul, Brazil; 3 Institute of Environmental, Chemical and Pharmaceutical Science, School of Chemical Engineering, Federal University of São Paulo (UNIFESP), São Nicolau St., Jd. Pitangueiras, Diadema 09913-030, São Paulo, Brazil; 4 Graduate School, Sciences and Engineering, National University of Trujillo, Av. Juan Pablo II S/N Urb. San Andrés, Trujillo 13011, La Libertad, Peru; 5 Federal Laboratory of Agricultural and Livestock Defense (LFDA-SP), Ministry of Agriculture, Livestock and Food Supply (MAPA), Campinas 70043-900, São Paulo, Brazil; 6 Technology and Research Institute (ITP), Tiradentes University (UNIT), Murilo Dantas Ave., Farolândia, nº 300, Aracaju 49032-490, Sergipe, Brazil; 7 Department of Pharmaceutical Technology, Faculty of Pharmacy of University of Porto (FFUP), Rua Jorge de Viterbo Ferreira, nº 228, 4050-313 Porto, Portugal; 8 REQUIMTE/UCIBIO, Faculty of Pharmacy, University of Porto, de Jorge Viterbo Ferreira, nº. 228, 4050-313 Porto, Portugal

**Keywords:** drug delivery, healing, medical applications, nanofibers, polymers, processing, rotary jet spinning (RJS), wound dressings

## Abstract

Wounds result from different causes (e.g., trauma, surgeries, and diabetic ulcers), requiring even extended periods of intensive care for healing, according to the patient’s organism and treatment. Currently, wound dressings generated by polymeric fibers at micro and nanometric scales are promising for healing the injured area. They offer great surface area and porosity, mimicking the fibrous extracellular matrix structure, facilitating cell adhesion, migration, and proliferation, and accelerating the wound healing process. Such properties resulted in countless applications of these materials in biomedical and tissue engineering, also as drug delivery systems for bioactive molecules to help tissue regeneration. The techniques used to engineer these fibers include spinning methods (electro-, rotary jet-), airbrushing, and 3D printing. These techniques have important advantages, such as easy-handle procedure and process parameters variability (type of polymer), but encounter some scalability problems. RJS is described as a simple and low-cost technique resulting in high efficiency and yield for fiber production, also capable of bioactive agents’ incorporation to improve the healing potential of RJS wound dressings. This review addresses the use of RJS to produce polymeric fibers, describing the concept, type of configuration, comparison to other spinning techniques, most commonly used polymers, and the relevant parameters that influence the manufacture of the fibers, for the ultimate use in the development of wound dressings.

## 1. Introduction

Chronic wounds affect patients suffering from different diseases and injuries (e.g., burns, trauma, or diabetes), becoming a major public and economic health problem worldwide due to high morbidity, risk of amputation, and severe socioeconomic burden [1,2,3]. When not treated properly, they negatively impact the patient’s quality of life. Besides an extended period for healing, they can lead to amputations and, in some cases, even death, depending on the injury [4]. In the United States alone, for example, more than 6.5 million suffer from chronic injuries, with treatment costs exceeding USD 25 billion a year [5].

Treatment of these lesions is currently accomplished through the use of autologous skin grafts applied onto the affected area to prevent pathogen invasion [6], which, in some cases, can even result in the amputation of the affected limb, as in the case of diabetic foot and severe infection. Such tissue injuries heal slowly, implying prolonged suffering for the patient and their families, requiring frequent wound management (cleaning, dressing replacement), in addition to high expenses for intensive hospital treatments and increasing risks of complications [1,7,8]. In consonance with this, more accessible solutions have been sought to find efficient and easy-to-manage therapeutic actions that reduce, prevent, and/or minimize wound aggravation, offering rapid healing in a functional and aesthetically satisfactory way. To meet this global need, several new materials and techniques are being proposed for the manufacture of wound dressings [9].

The demand for dressings that can be used to treat wounds has been growing and represents a significant impact on the global market (above USD 7 billion in 2020) with a forecasted growth at a compound annual growth rate (CAGR) of 9.7% by the year 2025 [10]. According to the National Wound Care Strategy Program (NWCSP, 2021), for each 2.2 million people who have a wound, 29% of them have acute wounds related to trauma, surgery, abscess, or burns requiring the use of wound dressings. These numbers drive the development of devices that can accelerate tissue regeneration in wounds, promoting rapid recovery of patients affected by chronic injuries, which are capable of restoring their life quality [11,12].

Rotary jet spinning (RJS) is a process for making membranes from a polymeric solution with scales ranging from micro to nanometric [13]. It uses a high rotational speed provided by an electric motor, dispersing the polymeric solution as a jet, quickly evaporating it, and depositing polymer fibers in a cylindrical collector [14,15]. This technique has advantages over other fiber methods, it is easy to operate and has high efficiency, requiring a low amount of solution to produce an extensive amount of fibers (high fiber yielding). Additionally, it does not require polymeric conducting solutions or a high voltage source, a fact that reduces the cost and enables the processing of various polymeric solutions without the need for a conductive solution [16,17,18,19]. Characteristics, such as flexibility, large surface area, and easy design modification, are being described for nanofibers obtained by RJS. The rotary-jet-spun fibers application field of the nanofibers obtained from rotary-jet-spun fibers can thus be used as filters, protective clothing, energy storage, sensors, and battery separators [20,21,22,23,24,25].

**Figure 1:**
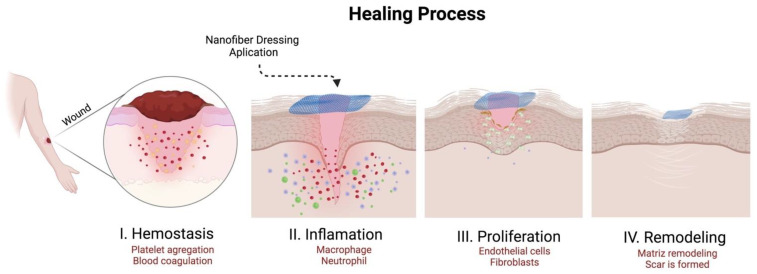
Scheme of the wound dressing action in the healing process (own drawing).

These RJS polymeric fibers are indicated for medical applications, especially in tissue engineering, drug delivery systems, and regenerative wound dressings [26,27,28,29]. Currently, polymeric membranes are used as dressings due to their high porosity (for oxygen permeation) and their large surface area (cell culture). It must maintain the humidity at the wound site and prevent the damaged tissue from particles and contaminants. The polymeric membrane should be non-toxic or allergenic, able to protect the wound from any trauma, impermeable to any bactericidal activity. It should have adequate thermal insulation, be comfortable, and be adaptable to the wound region, auxiliating the healing process (Figure 1) [30]. In addition, targeted drugs can be incorporated into the polymeric matrix, offering a therapeutic activity that collaborates in the healing process [31]. Some examples of RJS polymeric fibers used as potential wound dressings are made of natural polymers, usually with some pharmaceutical bioactive, such as the combination of poly (lactic acid)/gelatin/ciprofloxacin [32], carboxylated chitosan/polyethylene oxide/ibuprofen [33], and soy protein hydrolysate/cellulose [34], both presenting antibacterial activities, similar to the native extracellular matrix, potentially accelerating the skin regeneration.

There is currently a growing interest in rotary jet spinning studies because of its promising outcomes, especially in the use of RJS fibers for biomedical applications. In this review, we provide an in-depth discussion about the rotary jet spinning process regarding its technology and comparison to fiber methods, the influence of its operational parameters on the produced nanofibers, and how its products are applied in different areas, with a special focus on wound dressings, with some remarkable applications.

## 2. Spinning Techniques

Polymeric fibers can be produced by different techniques, e.g., electrospinning (solution and melt), melt blowing, drawing, and rotary jet spinning, among others. Indeed, some fiber production techniques have limitations regarding the produced fibers’ characteristics, equipment requirements, repeatability, productivity, and scaling. However, the RJS technique outstands itself as it produces high-quality fibers, is efficient and has easy scaling, has low energy consumption, and does not require conductive polymeric solutions [35,36,37]. However, a specific requirement for RJS is the use of a low boiling point solvent to evaporate at the operating conditions when the process occurs with a polymeric solution [38,39,40].

RJS is a technique that presents a simpler mechanism of operation and greater capacity for production concerning electrospinning [33,41]. In addition, the technique makes use of a smaller amount of solvents in the composition of the polymeric solution since the electrospinning the solution must be conducive as a prerequisite [13,19]. When compared to airbrushing, a technique that uses pressure difference for the film’s development, the production capacity of the RJS remains larger by the area of its collector, and also it does not require a high-pressure gas flow to promote the formation of polymeric fibers ranging from micro to nanometer scales [42,43].

To establish a general view of the fiber techniques, a comparison between the main techniques used for fiber production is presented in Table 1, summarizing the advantages, disadvantages, and applications of each mentioned technique.

## 3. Fundamentals of the Rotary Jet Spinning (RJS)

Rotary jet spinning (RJS) was first developed in 1924, with a USA patent, which proposed the generation of fibers using centrifugal force. In 2010, Badrossamay created the RJS machine as we know it today. Over the years, the technique has been improved according to numerous patents published, both in the USA and Europe [23,38].

**Figure 2:**
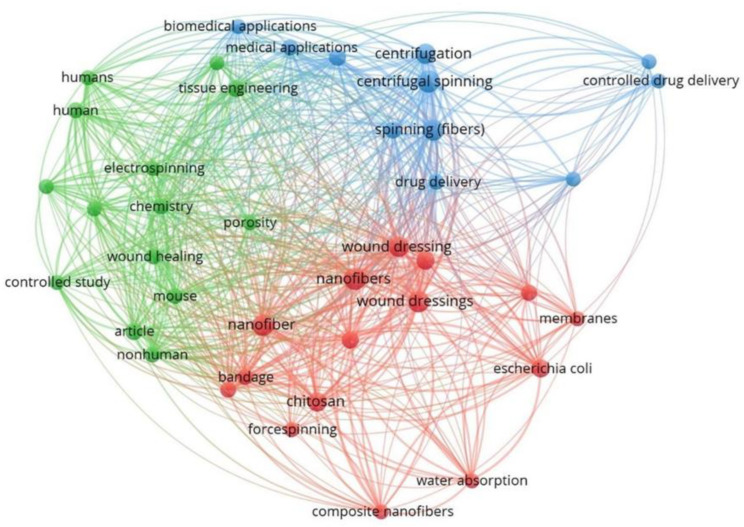
Publications about the RJS technology and the areas related to it in a bibliometric analysis performed by the free software VosViewer [65] with Scopus data.

From a bibliographic analysis carried out using the Scopus database (31 August 2022), searching the keywords “rotary jet spinning” or “centrifugal spinning” or “rotor spinning” or “pressure gyration”, which are common names for the RJS technique, 2651 documents were found. After this first screening, the keyword “wound dressing” was added, which is the focus of this review, and 25 occurrences were found between 2012 and 2022. Once the search was carried out in Scopus, the Vosviewer (version 1.6.18) software was used to build up the bibliometric map [64]. This software helps to visualize the bibliometric landscape of this theme, grouping the keywords related to the RJS technique into three clusters (Figure 2). The red group has the majority of the keywords (wound dressing, nanofibers, membranes, biocompatibility, bandage, and chitosan), followed by the green cluster (humans, tissue regeneration, electrospinning, and wound healing) and the blue cluster (centrifugal spinning, centrifugal, spinning (fibers), biomedical applications, and drug delivery). From Figure 2, we confirm the great demand for the nanofibers produced via the RJS technique, also showing its notorious research in the biomedical field, and wound dressing fabrication.

The process involved in the RJS technique is considered simple, however, some knowledge of polymer chemistry, processing, and fluid mechanics is the basis for a proper understanding of this technology. Despite the operating conditions, the polymers’ intrinsic properties (viscosity, concentration, molecular structure, molar mass, and surface tension) also affect the fibers’ properties [39,40,66,67,68].

**Figure 3:**
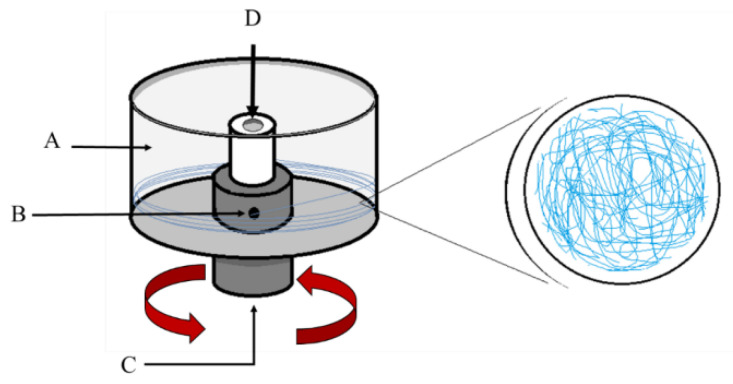
RJS apparatus: (**A**) collector, (**B**) reservoir, (**C**) motor, (**D**) feeding zone (modified with permission from [69], Copyright 2015 American Chemical Society).

A simplified scheme of the RJS technique is presented in Figure 3, in which the main components that form the configuration of the RJS system are illustrated.

The RJS equipment is composed of a reservoir, a spinning head, that can have two opposing orifices (syringes) in the body (nozzle-type) or just have a cylindrical shape (nozzle-less type), and is connected to the shaft with a controllable rotation speed motor [23]. In the RJS technique, the structures obtained (nanofibers) depend on the action of centrifugal force, just like in a cotton candy machine [70,71]. Due to the centrifugal force generated by the motor action, a polymeric jet is ejected from the reservoir orifices. This occurs when the centrifugal force and hydrostatic pressure surpass the capillary resistance of the solution/polymer (related to surface tension and viscosity), with the polymer extrusion elongating and thickening with the rotation speed [72]. In the centrifugal spinning process, nanofibers can be obtained from a polymer solution or melted polymer, and the diameter and morphology of the fibers depend on the diameter and length of the ejector nozzle [73].

**Figure 4:**
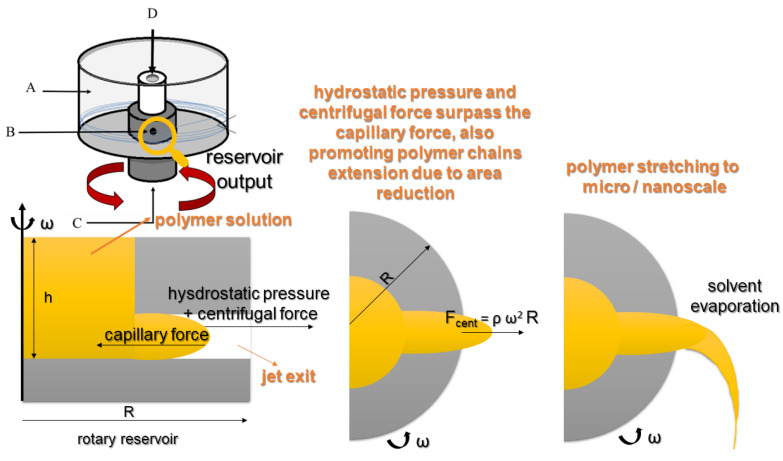
Polymer molten jet formation in the RJS apparatus (modified with permission from [74], Copyright 2018, IOP Publishing Ltd.).

The polymer jet behavior is depicted in Figure 4, where it is possible to observe that the fluid is a jet from the spinneret as the minimum angular velocity is achieved, forming a spherical front end due to surface tension. As the rotating disk velocity increases, the polymer fluid forms a conical droplet, which is stretched if the centrifugal force surpasses the capillarity acting in the polymer (viscous force), forming the micro/nanofibers [20,74,75]. The solvent evaporation is enhanced according to the polymer jet traveling in a spiral, forming fibers of extremely small diameters, implying greater surface areas; at the same time, the solidification process also occurs [14,21,36,39,76]. Finally, the polymer fibers settle in the collector’s base due to the action of gravity.

### 3.1. Melt RJS

**Figure 5:**
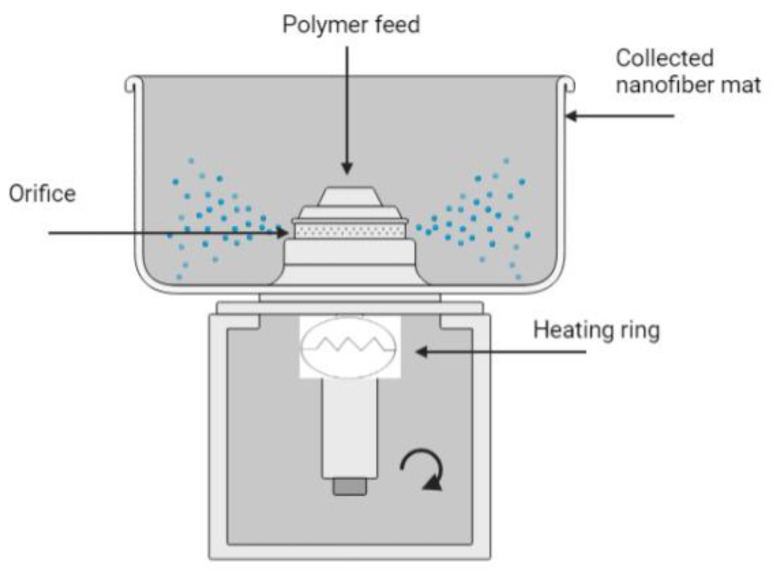
Melt RJS scheme (modified with permission from [72], Copyright 2012 American Chemical Society).

Melt RJS uses molten polymer in the RJS apparatus to form micro and nanofibers without using solvents, which is important for special polymers that are hard to dissolve in common organic solvents [73]. Under this condition, it usually employs high temperatures to melt the polymer, thus, the molten polymer drives into the orifices of the rotating spinneret [72]. Additional components that help the processability rely on viscosity-reducing additives or plasticizers which improve the fluidity and allow the production of fibers with reduced diameters [77]. The polymer jet is then elongated according to the centrifugal force and solidifies at room temperature, yielding micro/nanofibers at the collector (Figure 5). Some drawbacks here include the polymer fluidity because the fibers of the high viscous molten polymer need to elongate without bearing and breakage during the spinneret extrusion to produce quality fibers.

### 3.2. Immersion RJS

**Figure 6:**
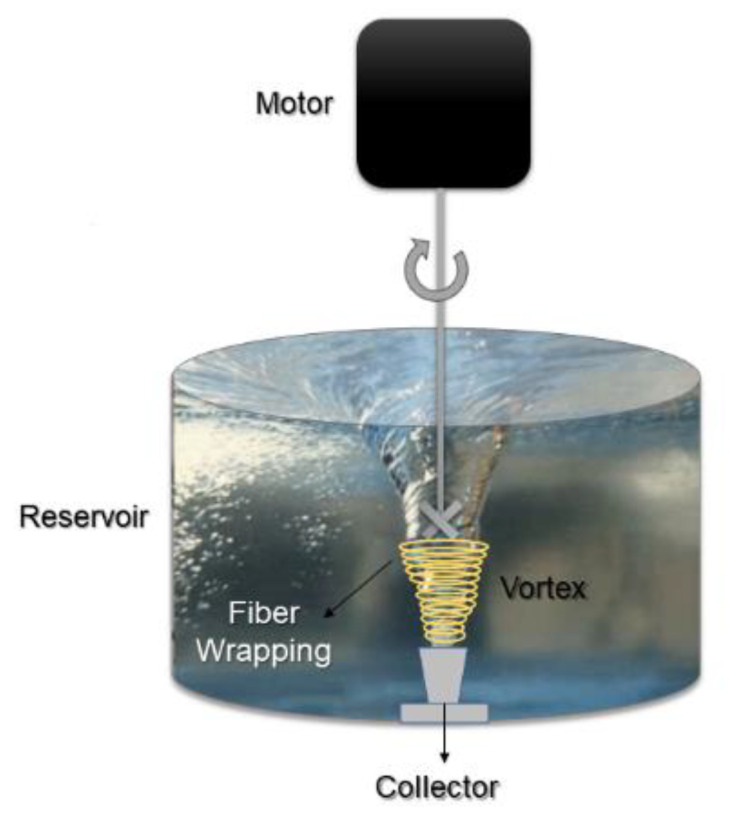
Immersion RJS scheme (modified with permission from [78], Copyright 2017 John Wiley & Sons).

The immersion RJS (Figure 6) consists of dropping the polymer extruded into a solution where the fiber solidification and/or crosslinking occurs, which is advantageous since it minimizes extrusion breakage and bearing in the fibers due to reducing the surface tension [78]. As the polymer fibers are deposited in a liquid, it does not need a volatile solvent in the process since the fibers solidify in this liquid, or crosslinker agents can be present in this bath. Thus, some precautions need to be taken in this process, for example, the immersion liquid cannot be water for hygroscopic polyesters or polyamides since they can suffer hydrolysis, affecting their properties. Various polymers and solvents were studied by Gonzalez et al. (2017) to demonstrate the immersion RJS viability for processing different polymeric systems. The crosslink effectiveness of the immersion RJS to produce fibrous gelatin employing a bio-crosslinker (enzyme) resulted in promising 3D aligned fibers for cell support, useful for food processing (future meat) [79].

### 3.3. Nozzle-Less RJS

**Figure 7:**
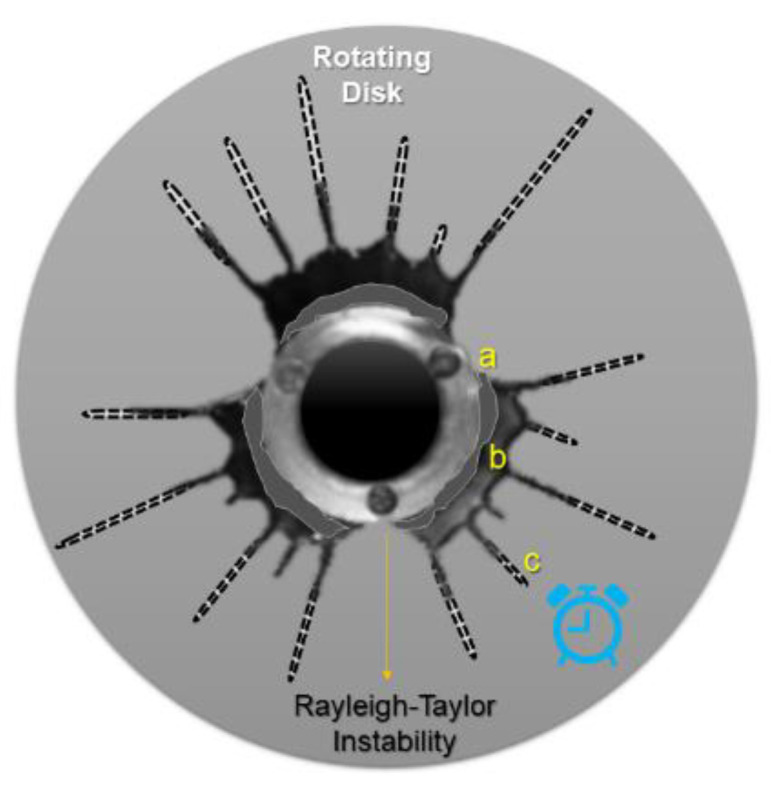
Polymeric finger prints from nozzle-less RJS increasing with processing time (modified with permission from [37], Copyright 2014 Elsevier).

Nozzle-less RJS does not use a needle for the polymer extrusion; instead, it pulls out of a lid-disk gap in the center of a rotating disk collector, thus the jet liquid polymer produces “fingers” due to Rayleigh–Taylor instability (Figure 7). Such a method is an alternative to nozzle RJS systems that are susceptible to high viscous polymer clogging of the needle orifice [37].

## 4. Parameters and Factors Influencing Rotary Jet Spinning

RJS technology is a prospective method to manufacture high-performance three-dimensional nanofibers with uniform morphology, high efficiency, and productivity. In this process, there are key parameters that control the fiber properties (diameter, mechanical strength, morphology, porosity, etc.), including polymer concentration, rotational speed, nozzle diameter, orifice-to-collector distance, and solvent volatility [23,40,71,80,81,82].

Besides the attractiveness and advantages of the RJS technique, defects might occur in the polymer fiber during its processing, usually caused by Rayleigh instability (driven by surface tension), forming beads, and defects that diminish the fiber’s superficial area [83]. Solvent rate evaporation and rotational speed might control the polymer fiber solidification, driving the smoothness and/or fibers’ porosity, besides the airflow resistance also influencing the fiber elongation [75].

Depending on the RJS type, certain intrinsic properties and operating conditions directly influence the fibers’ production (Table 2). For this reason, the RJS parameters must be studied and optimized to ensure that the resulting fibers have all the required properties for their targeted application, especially in the biomedical area, which needs restricted characteristics.

Additionally, the RJS process might have new variants in the production system. It is a hybrid RJS process that combines other acting forces besides the rotary ones to improve the manufacturing of the fibers. According to this concept, electrostatic-centrifugal spinning and photo-centrifugal spinning additionally use electrical force [98] and photoinitiators with the incidence of UV lights [97], respectively.

## 5. Biomedical Applications of RJS-Nanofibers

RJS nanofibers are widely used in the biomedicine field since these fibers have interesting characteristics that are required in this area [13,66]. Therefore, caring for skin lesions (wounds) is not a simple treatment since it requires the protection of the injury from the action of external physical, mechanical, or biological agents, to reduce, prevent, and/or minimize the risks of resulting complications (e.g., secondary infections).

The main requirement for a polymeric wound dressing is biocompatibility. This means that interactions between biomaterials, cells, and the host must not be potentially harmful to induce cytotoxicity, generate adverse immune responses, or activate coagulation pathways [99]. Concerning the use of biomaterials for cutaneous wound healing, some characteristics are essential: ease of handling and application to the wound site; to be readily adherent; exhibit adequate physical and mechanical properties; have a controlled degradation; to be sterile, non-toxic, and non-antigenic; exhibit minimal or no inflammatory reactivity; to be incorporated into the host with minimal scarring and in a painless manner; and to facilitate angiogenesis [2]. In the case of using dressings, these must have a highly porous structure and at the same time act as a water barrier to keep the wound moist, accelerate healing, and prevent bacterial invasion, which can be caused by a fluid accumulation between the wound and the dressing [100]. RJS nanofibers have high surface areas (porous films), promoting conditions for cell growth and wound repair [36,101]. Additionally, their structure imitates the natural skin interface, allowing the recovery of biological functions of damaged tissues and selectively interacting with specific cell lines.

The mechanical properties (strength, modulus, toughness, and ductility) and architectural properties of the wound dressings are also extremely important so that they are not compromised during their use. Therefore, the dressing cannot be completely solid, because, in addition to porosity and interconnectedness, permeability is also an important material characteristic, as it is a measure of liquid fluidity in the structure. Thus, high permeability produces greater diffusion within the membrane, facilitating the entry of nutrients and removal of degradation products and metabolic wastes [100].

Some biomedical applications of RJS nanofibers are presented in Table 3.

There are several advantages to using nanofibers to make drug-releasing wound dressings, mainly because these materials can protect the wound, absorb exudates, provide better penetration of the drug into the wound bed, increase intracellular uptake, reduce toxicity, reduce the frequency of topical application and increase patient compliance, and accelerate the healing process [116,117,118]. In addition to drug delivery, nanofibers can be a promising tool for the incorporation of macromolecules, antibiotics, anti-inflammatory, growth factors, enzymes, or nucleic acids due to their specific delivery capacity allowing high bioactivity due to controlled release [119,120].

**Figure 8:**
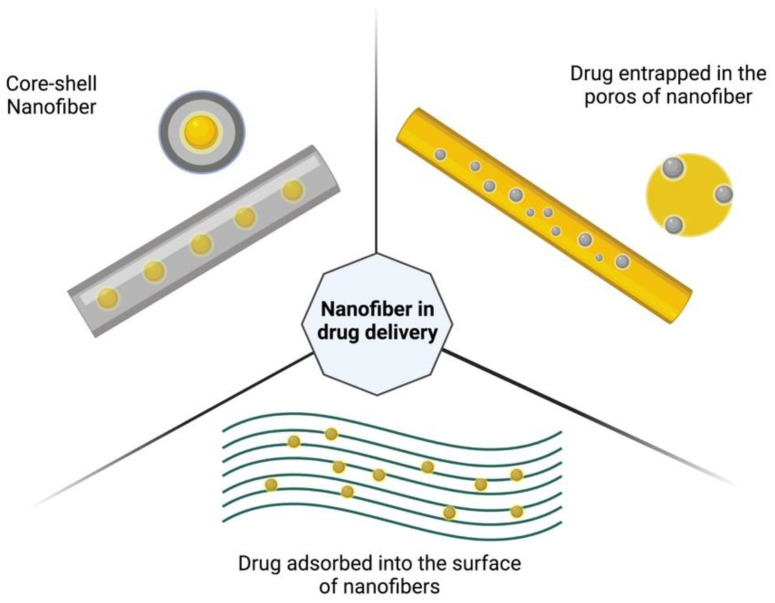
Structures of different designs in the fabrication of nanofibers for drug delivery (created using Biorender—https://biorender.com/ (accessed on 9 august 2022)).

Factors such as geometrical properties, diameter, specific surface area, and total pore volume affect the convection and diffusion of the liquid in which the nanofibers are immersed, thereby affecting the drug release properties, which in turn affects the understanding of the solid state of the drug and the effect of polymers incorporated into the nanofibers on drug release. The study of kinetics and its mechanism is extremely important for drug delivery systems, as is the nanofiber design (Figure 8). In nanofibers with a homogeneous structure, the drug is distributed in a polymer matrix, unlike what occurs in core-shell nanofibers, where the drug-carrying matrix is covered by a polymer shell, or nanofibers can be developed, on which the drug can be immobilized on its surface [121,122,123].

A poly(L-lactic acid) (PLLA) membrane incorporated with curcumin produced by the RJS technique showed a controlled release profile justified by the low rate of fiber degradation. In addition, it presented biocompatibility and non-toxicity against fibroblasts, giving the membrane a potential material for the treatment of chronic wounds [31].

Core-shell nanofibers were designed to control the release rates of ibuprofen and human epidermal growth factor (EGF) during the inflammatory and proliferative phases of wound healing by coaxial centrifugal spinning technology [33]. To prepare core-shell nanofibers, 10 mg/mL ibuprofen and 1 µg/mL EGF were mixed into a 1:1 solution of carboxylated chitosan (CCS), polyethylene oxide (PEO). It was stirred on ice for 15 min. The rotary spinning system had a 4500 rpm speed with a 30 cm collection distance. The release results showed that the CCS/POA core-shell nanofibers containing EGF showed a release rate of 75% in two hours and thereafter maintained a controlled release rate of the remainder of the growth factor. It was also observed that the release of EGF in the central layer of the core-shell nanofiber was moderate due to the active agent incorporation in the central layer. The diameters of the core-shell nanofibers and uniaxial nanofibers are 1154 nm and 418 nm, respectively, which means that the erosion rate of the core-shell nanofiber polymer is lower compared with that of the uniaxial nanofibers, which directly affects the EGF release. For ibuprofen-containing core-shell nanofibers, the release rate was greater than 50%, but for the monoaxial nanofibers, the rate was only 30%. This result may be related to the different initial positions of ibuprofen in the core-shell and monoaxial nanofibers, leading to the polymer erosion mechanism. These outcomes demonstrate a promising application of nanofibers in the delivery of drugs in wound dressings.

An antimicrobial dressing formed by poly(lactic acid) (PLA)/gelatin/ciprofloxacin (CPF) nanofibers manufactured by the RJS was developed [32]. At first, it was observed that different concentrations of the prepared solutions influence the diameter of the nanofibers. The results were shown for concentrations of 0–12% of ciprofloxacin, the nanofibers had diameters ranging from 513–622 nm, respectively, and this result was associated with the chain solution, increasing nanofibers. The release profiles showed that the PLA/GE nanofibers released about 30% of ciprofloxacin in 1h; this result was attributed to the CPF adsorbed on the nanofiber surface. After this time, all nanofibers with different concentrations of CPF maintained a slow and sustained release due to the control mediated by the PLA polymer chains. The release of nanofibers reached 87% at 144 h in particular for formulations containing 10% and 12% CPF. With these results, it was possible to observe that the process of developing the nanofibers was satisfactory mainly because it presents a controlled drug release system, providing a promising dressing for treating wounds.

Odermatt et al. [124] patented the method that describes the creation of nanofibers containing at least one synthetic and bioabsorbable polymer in the form of a dressing. Despite the already existing healing devices on the market, some disadvantages such as the occurrence of infections, irritation, and material degradation lead to the need for the development of new dressings. Through the RJS technique, researchers seek a greater effectiveness of this material in the healing process as well as to develop an environmentally friendly product with low process energy. Different synthetic polymers such as polycaprolactone, polylactide-co-glycolide, polyvinyl alcohol, hyaluronic acid, acetylated distarch phosphate, and starch were used for the development of these dressings in different concentration gradients. Thicknesses between 30 nm and 400 nm were obtained through the different mixtures of polymers used to manufacture rotospun fibers. Despite recent studies demonstrating the effectiveness and potential of manufacturing nano- and microfibers by RJS in the development of new dressings, the need to advance new studies aims to bring to the market a new product that has greater effectiveness, low cost, and easy production for the treatment of wounds.

In comparison with other existing methods of producing nanofibers, the RJS technique has an enormous potential to be explored, mainly because it provides high yield at a low cost and can be used to carry drugs in nanofibers for the production of dressings that provide the patient a product by maintaining a controlled release, protecting the active agent, reducing antimicrobial resistance events, and consequently accelerating the healing and restoration of the affected area.

## 6. Other Applications: Filters and Batteries

Filters are a remarkable industrial application of polymer nanofibers produced via the RJS technique. Such a process is ideal for the production of continuous fibers since it ensures a high surface/volume ratio of the fibers, which efficiency increases with this ratio. Accordingly, polyamide 6 (PA6) presented smaller fiber diameters with RJS than the electrospinning process, for a PA6 (22.5 wt.%) solution containing formic acid, confirming that RJS quickly creates fibers at the nanoscale and proving that RJS is adequate for industrial production of polymer nanofibers [19].

RJS air filters compete with typical high-performance filters (HEPA—high-efficiency particulate air). Normally, an air filter must have 99.97% of minimum removal efficiency for particles equal to or greater than 300 nm in diameter, according to the standardization of the United States Department of Energy, or 85–99.9% particle removal, as Europe standardizes (European standard EN 1822: 2009). Additionally, it also requires an airflow rate between 3 and 10 m^3^·s^−1^, and approximately 300 Pa minimum filter pressure drop [36]. Fiber-based filters have a medium-low price range, but, with RJS, a scalable and high-productivity technique, it is possible to maintain the market prices with the same efficiency as HEPA filters.

The main function of air filters is to prevent the users from health problems that particulates can offer [125]. As a consequence, studies have shown that nanofiber-based filters present higher particle removal efficiency than filters built up with greater diameter fibers. For example, a mining vehicle containing cellulose-based filters exhibited a dust reduction from 86 to 93% when using nanofibers in the filter composition [126]. Likewise, the quality factor (efficiency and pressure drop) is reported to increase at most 2.6 times in nanofiber-based filters compared to those made with microfibers [127]. Additionally, the COVID-19 pandemic boosted the use of the RJS technique for the production of filters for facial masks to protect the population from the mutating virus [128].

Multilayer electrospun nanofibrous membranes formed by Polyvinylidene Fluoride (PVDF)/Polyacrylonitrile (PAN) achieved high filtration efficiency and low pressure drop [129]. This is because PVDF has a greater porosity, which is adequate for air passaging, thus, reducing the pressure drop, while PAN has the characteristic of capturing small particles. Additionally, poly [2-(N, N-dimethyl amino) ethyl cationic methacrylate] (PDMAEMA) was added to the PVDF solution. Thus, it resulted in a composite air filtration system for biological protection, with excellent antibiotic removal, reaching a high inhibition rate (≈90%).

Regarding water filtration, one of the main concerns is about the capture of heavy metals in wastewater, since these metals are toxic, representing problems for human health and the environment as well [130]. For this reason, some studies with cellulose acetate membranes proved that this material is very efficient for nanofiltration due to the regeneration of pure cellulose via deacetylation [131]. Cellulose acetate membranes displayed decent adsorption and desorption after being washed five times with a sodium hydroxide aqueous solution, and also presented an absorption capacity of 19.5 mg/g according to the Langmuir isotherm [130]. Additionally, cellulose nanofibers embedded with polyamide layers via interfacial polymerization resulted in a filtration membrane with Mg^2+^ rejection (MgCl_2_ and MgSO_4_), depending on the extension of the interfacial polymerization [132].

Besides the filtration area, fibers produced by the RJS technique have also been applied as photovoltaic fibers [36,133]. Polymeric materials such as inorganic semiconductors contributed to this application since it combines ease of processability, compatibility, and low-temperature manufacturing processes. Such materials also enable large-scale production, allowing the development of lighter and more flexible devices [134,135].

In the field of photovoltaics, power conversion efficiency (PCE) can be improved with the heterojunction of materials [136]. Likewise, high-performance cells are directly related to the morphology of the active layer [134], thus adding a rich-concentrated nanofibers solution to photovoltaic cells still increases PCE [137]. Additionally, with a solution containing nanofibers and a molecular acceptor, such as [6,6]-phenyl C61-butyric acid methyl ester (PCBM), it is possible to obtain a high-efficiency active layer for organic solar cells with a PCE up to 3.6% [133]. The study by Burson et al. (2007) concluded that the photovoltaic effect may require disorganized polymer portions to fill the gaps present in the nanostructured layers and ensure contact between the donor fibers and the PCBM acceptor domains [133]. Other interesting applications for RJS fibrous membranes and their composites are Li-ion battery separators and proton exchange membrane (PEMs) fuel cells [138]. An example is polyvinylidene fluoride (PVDF) nanofibers semi-interpenetrated with perfluorosulfonic acid (Nafion^®^) to develop PEMs [139], in which the PVDF crystalline structure, specifically its β-phase, increases the PEM’s piezoelectric performance [140,141].

## 7. Conclusions and Future Prospects

Recent developments in nanofiber fabrication technology have led to controlled manipulation of nanofiber properties, such as high surface area/volume ratio and their ability to encapsulate bioactive molecules for controlled release. Factors such as increasing awareness of advanced wound healing techniques and increasing incidence of ulcers due to obesity, diabetes, cardiovascular, and degenerative diseases are driving the growth of the wound care market. RJS technology is a relatively new process for producing polymer fiber material systems for biomedical applications, which can be used to rapidly produce large quantities of final products. It can also create biomimetic porous extracellular matrix structures (scaffolds) that allow for biocompatibility, cell adhesion, and proliferation. Additionally, biopolymeric nanofibers from the RJS process have the versatility to provide materials with tunable mechanical (tensile modulus, peak load, and break strain) and biological properties (biodegradability, air diffusion), aiding tissue regeneration technologies. Their high porosity provides better diffusion of oxygen and pharmaceutics, which is greatly advantageous for epidermal applications, especially for wound dressings. These characteristics turn the biopolymeric nanofibers into a powerful building block for designing more comprehensive pro-regenerative solutions. The production of RJS polymer scaffolds results in materials with excellent properties, combining processability, strength, elasticity, and biocompatibility. Besides the advantages of the RJS technique, it is expected that the advancement of this technique will lead to customizable healing formulations, as there are several skin injuries (burn, trauma, surgery, diabetic ulcer, infection, etc.) that require targeted treatments for wound healing. RJS wound dressings are still in progress in the market, but their promising features for fast healing, drug delivery, and improvement in the quality of patients’ lives with less invasive procedures are attracting attention and popularity for the RJS technique. With the RJS technique, it is possible to produce dressings using a smaller feed of raw material in comparison to other known production techniques, however, the dressings are customized without a unitary standard for each clinical case, so it is still necessary to explore such conditions and invest in inputs to serve this niche uniformly. One obstacle that must be overcome in the use of RJS for wound dressing production involves its production capacity, as there is no record of industrial-scale RJS equipment.

## Figures and Tables

**Table 1:** Spinning techniques, their main advantages, disadvantages, and applications.

Techniques	Advantages	Disadvantages	Applications	References
Electrospinning	Nanometric fiber diameters (100–1100 nm), large surface area, uniform and aligned fibers, high porosity, simple fabrication, superior mechanical properties, and ECM-like structure.	Requires a high voltage source and conductivity solution, uses toxic solvents, has low productivity, has difficulties in scaling and equipment handling.	Biomedical: regenerative medicine and drug delivery systems. Others: electrochemistry (Li-air battery separator), catalysis (sensors), photocatalysis (organic solar cells), and environmental (filters).	[44,45,46,47,48,49,50]
Melt Blowing	Long and continuous fibers, high productivity, solvent-free.	High temperatures, thermal degradation, larger fiber diameters, and polymers limitation due to viscosity control.	Textile area and filters.	[51,52,53,54,55,56]
Drawing	Simple process, high repeatability, produces unique, continuous, and long nanofibers.	It uses viscoelastic materials. Limited to laboratory scale, it is a discontinuous process.	Agriculture packaging.	[14,51,55,57]
Rotary Jet Spinning	Process easy to scale, good repeatability, fiber dimension control, free from high voltage, low cost, simple operation, eco-friendly. Numerous polymers can be processed, besides polymeric emulsions and suspensions, with high productivity.	Might require high temperatures. Larger diameter fibers. Fiber properties can be affected by the material’s characteristics and quality/configuration of RJS equipment.	Controlled drug release, wound dressings, tissue engineering, aerosol filtration, energy storage, edible films, nutraceuticals, food encapsulation, and packaging.	[14,31,51,53,58,59,60]
Air Brushing	Uncharged solution, fibers diameter controlled by air pressure and nozzle diameter, coating various shapes, fast deposition rates.	Highly viscous polymer solutions are difficult to produce fibers, require compressed air, and solvent evaporation depends on the solvent itself.	Scaffolds, tissue engineering, filtration.	[61,62,63]

**Table 2:** Parameters that affect the properties of the RJS resulting fibers.

RJS Type	Sub-Type	Characteristics	References
**Traditional RJS**	Melt Spinning	Polymers are melted and extruded Melt viscosity, temperature, rotational speed, and orifice sizes impact this process	[84]
			[85,86]
	Polymer Solution Spinning	↑ polymer molar mass → ↑ jet diameter ↑ solvent vapor pressure → ↑ uniform and ↓ porous fibers ↑ surface tension → ↑ beads formation ↑ polymer concentration → ↑ fiber diameter ↓ polymer concentration → ↑ fiber diameter Polymer concentration must be optimized to ensure uniform and thin fibers Rotational speed highly impacts the ideal concentration ↓ solution viscosity → ↑ beads formation To produce ideal fibers, viscosity must be low (1–10 Pa.s), presenting Newtonian behavior ↑ rotational speed → ↓ fiber diameter ↓ nozzle diameter → longer fibers Straight nozzle angle → ↓ fiber diameter Distance spinneret—collector → depends on solvent evaporation and fiber formation	[87] [75,83,87,88,89,90,91] [75,83,88,92] [74,88,92,93,94] [74,88] [91] [74,88] [75,83,90,91] [27] [83,94,95] [92] [94] [75,91]
**Hybrid RJS**	Electrostatic-Centrifugal Spinning	Centrifugal and electrical forces are combined to improve the fibers This association enhances the fibers’ rheology because it removes “whipping instability”	[96]
	Photo-Centrifugal Spinning	Involves the use of photoinitiators and UV lights to produce fibers Depends on a high effective light intensity, polymer concentration, and time exposure to develop uniform fibers	[97]

**Table 3:** Recent developments in polymeric nanofibers produced by RJS in the biomedical area.

Polymers Used	Applications	Characteristics	References
Biological ECM ^1^/HA ^2^	Tissue engineering	These scaffolds of porous nanofibers produced by iRJS ^3^ have tunable properties, as they are composed of biological molecules (HA, fibrinogen, collagen, gelatin, and chondroitin sulfate) that biomimics the ECM to speed up tissue regeneration	[102]
CS ^4^/PEO ^5^	Tissue engineering	Fabrication of continuous, ultrafine, and uniform beads-free nanofibers with high CS content for enhanced antimicrobial and biocompatibility	[103]
OCS ^6^/TOB ^7^	Tissue engineering	OCS grafted with an antibiotic (TOB) was processed with PEO in a RJS equipment, such polymer improved the spinnability, with the formulation 1:3 OCS-TOB/PEO showing the best antibacterial activity	[104]
PCL ^8^	Bone regeneration	PCL scaffolds combined with nHAp ^9^ produced via RJS were used in bone structures. The results showed that the PCL/nHAp scaffolds had a positive influence on the flexural mode of the newformed bone	[105]
PCL	Tissue engineering	This study demonstrates that RJS-spun fibers have a unique morphology compared to electrospun fibers, are non-cytotoxic when in contact with mammalian cells, and reduce bacterial colonization without the need for further incorporation of antibiotics or prior chemical treatment	[39]
PCL/Gelatin	Tendon tissue engineering	Dual-phase fibers have been developed involving RJS and WES ^10^ techniques. The fiber core is formed by gelatin, presenting adequate mechanical strength, and also helping the tendon osteogeneses	[106]
PCL/Gelatin	Tissue engineering	RJS proved to be effective to produce non-toxic PCL-gelatin fibers that possibly allow their use as scaffolds	[107]
PCL/nHAp	Orthopedic applications	Scaffolds with PCL/nHAp showed reduced bacterial proliferation in bones (in vitro and in vivo) since the structures obtained presented superhydrophobic behavior	[108]
PCL/β-TCP ^11^	Bone grafting	PCL and β-TCP were solubilized in chloroform, and further spun at 3500 rpm, where formulations M5 and M10% promoted better collagen and osteoclasts production	[59]
P4HB ^12^/Gelatin	Scaffolds for heart valve replacement	The hybrid fibers (core—gelatin, exterior—P4HB) produced a biomimetic fibrous matrix, such as heart valves, improving the regeneration of the fibrous tissue	[109]
PLA ^13^	Bone tissue engineering	PLA/SBA ^14^-15fiber improved polymer matrix biocompatibility and osteoblast cells’ adhesion	[110]
PLA	Tissue engineering	Polymeric roughened microfibers (PRM ^15^), with high porosity, produced by RJS, improved the mesenchymal stem cells’ adhesion and tissue incorporation, reducing the stroke lesion area	[111]
PLLA ^16^	Tissue engineering	Fibrous PLLA membranes produced by the RJS technique had non-toxic behavior, presenting biocompatibility and bioadhesion, which makes them adequate support for fibroblastic and osteoblastic cells’ proliferation	[15]
PU ^17^	Tissue engineering	The PU fibrous structures produced by RJS, both aligned and random, showed compatibility with the cultured osteoblastic cell line, which allows its application in tissue engineering	[112]
PU	Tissue engineering	PU scaffolds combined with collagen and elastin showed an absence of solvent in the fibers, besides hydrophilic behavior, which possibly allows their application as tubular scaffolds for regeneration of vascular systems	[113]
PVP ^18^	Biomedical	The compact equipment easily controlled the operating parameters, producing aligned and homogeneous PVP fibers suitable for drug delivery systems	[22]
PCL	Tissue engineering	Fabrication of scaffolds with micro and nanofibers of polycaprolactone and gelatin for the cultivation of cardiomyocytes for a biofabrication of ventricles	[114]
PLA/PCL	Biomedical	Dressing fibers produced by RJS containing polymeric fibers incorporating VANC ^19^ were developed in order to evaluate the antimicrobial potential against *Staphylococcus aureus*	[115]

^1^ ECM—extracellular matrix; ^2^ HA—hyaluronic acid; ^3^ i-RJS—immersion rotary jet spinning; ^4^ CS—chitosan; ^5^ PEO—polyethylene oxide; ^6^ OCS—oxidized chitosan; ^7^ TOB—tobramycin; ^8^ PCL—polycaprolactone; ^9^ nHAp—nanohydroxyapatite; ^10^ WES—wet electrospinning; ^11^ β-TCP—tricalcium phosphate; ^12^ P4HB—poly-4-hydroxybutyrate; ^13^ PLA—polylactic acid; ^14^ SBA—silica mesoporous; ^15^ PRM—polymeric roughened microfibers; ^16^ PLLA—poly(L-lactic acid); ^17^ PU—polyurethane; ^18^ PVP—polyvinylpyrrolidone; VANC—Vancomycin ^19^.
